# Supplementary material for: Dietary Intake of Green Nut Oil or DHA Ameliorates DHA Distribution in the Brain of a Mouse Model of Dementia Accompanied by Memory Recovery
Source: Nutrients. 2019 Oct 4;11(10):2371. doi: 10.3390/nu11102371 (PMC6835595; doi:10.3390/nu11102371)
Supplement: Supplementary file 1 [file nutrients-11-02371-s001.zip › Supplementary files/Supplementary_Materials.docx]

**Supplementary Materials**

**
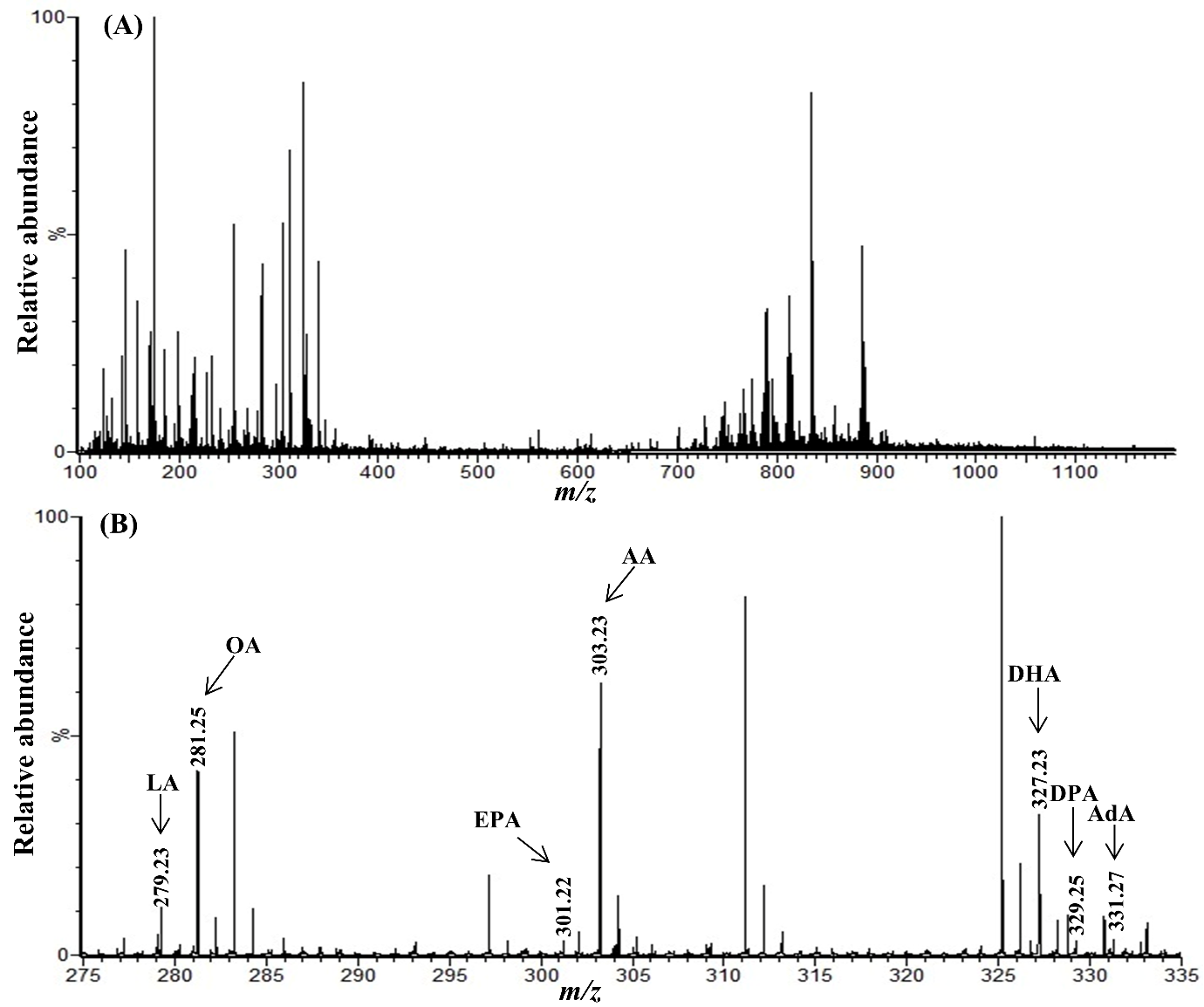
**

**Figure S1:** Representative negative ion DESI mass spectrum from sagittal slices of SAMP8 mouse brains and detection of *m/z* of interest (7 *m/z*). (A) Full mass spectrum. (B) Expanded view of A. LA, OA, EPA, AA, DHA, DPA, and AdA indicate linoleic acid, oleic acid, eicosapentaenoic acid, arachidonic acid, docosahexaenoic acid, docosapentaenoic acid, and adrenic acid, respectively.


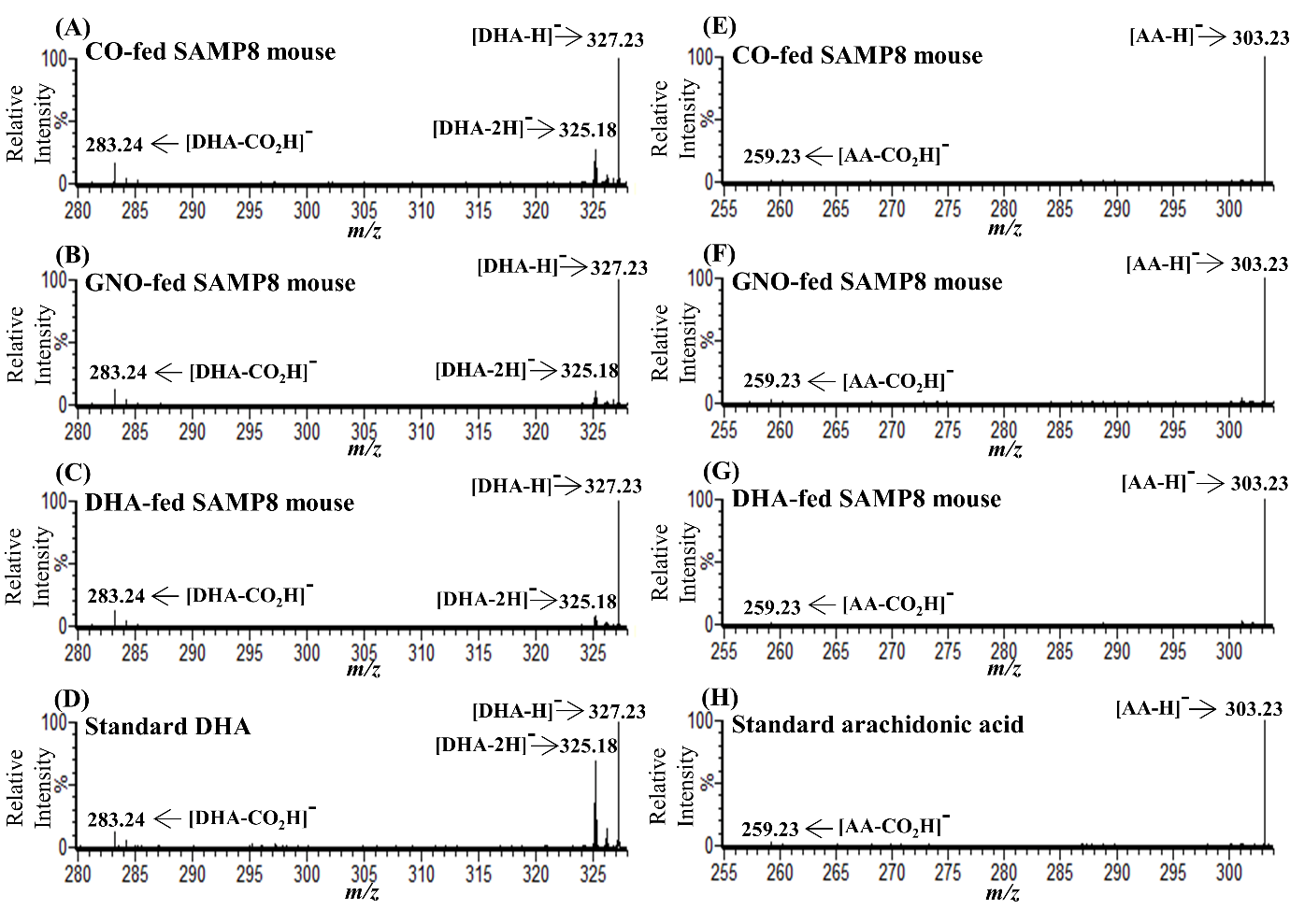


**Figure S2**: DESI-MS/MS mass spectra of *m/z* 327.23 (A-D) and 303.23 (E-H) in negative ion mode. Similar fragments were detected in both samples and standards.

CO, GNO, DHA, and AA indicate corn oil, green nut oil, docosahexaenoic acid and arachidonic acid, respectively. For DESI MS/MS, data was acquired using the following parameters: collision energy of 10 eV, source temperature of 120°C, capillary voltage of 4.0 kV, 98% methanol (98:2; methanol: water) at a flow rate of 2 µL/min, mass tolerance of 0.05 Da, and N_2_ gas pressure of 0.4 MPa.

**
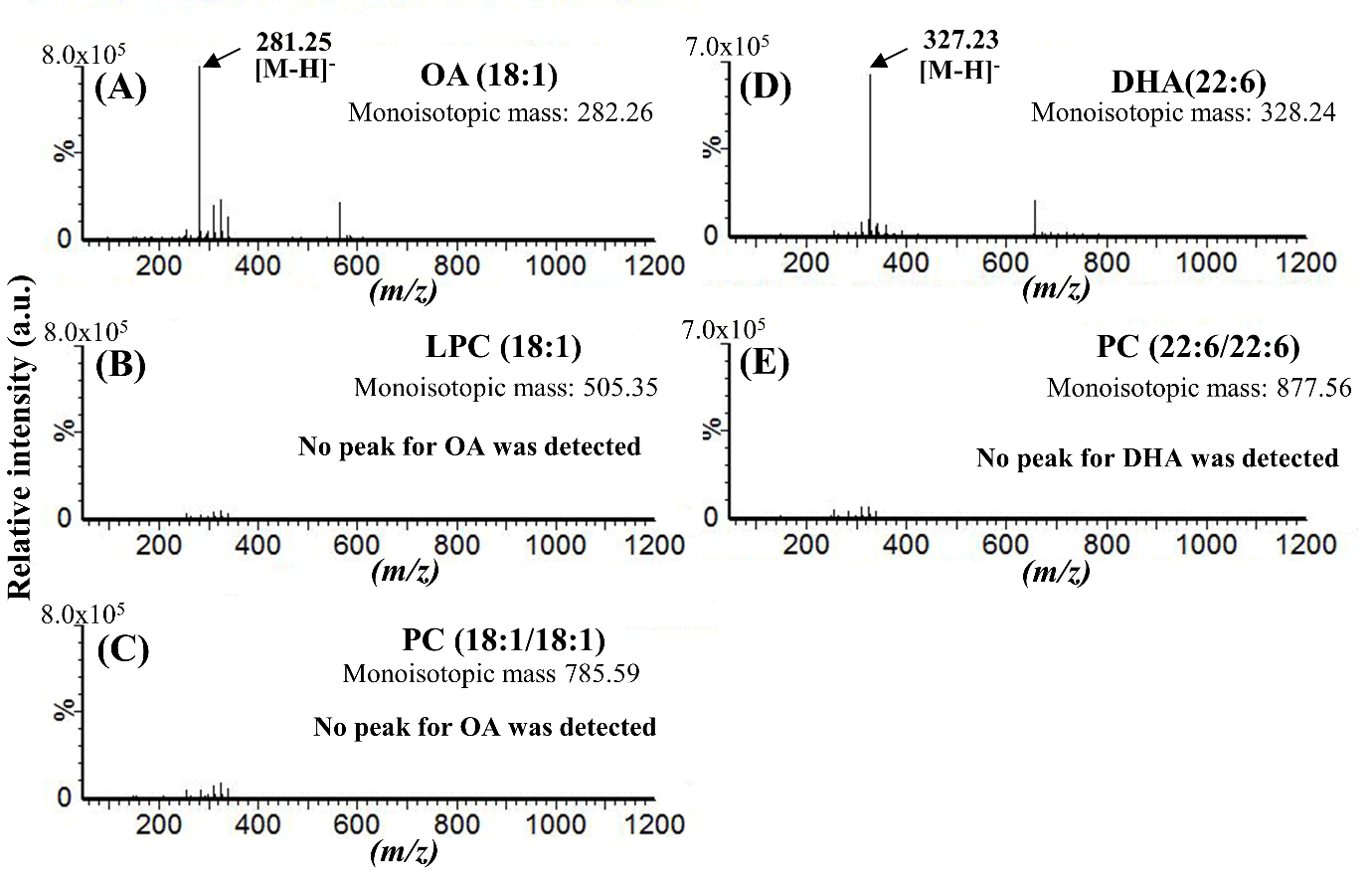
**

**Figure S3**: DESI-IMS mass spectra in negative ion mode from five lipid standards, OA (A), OA containing LPC (B), OA containing PC (C), DHA (D) and DHA containing PC (E).

Data from lipid standards were acquired using same instrument and same parameters which were used to acquire data from SAMP8 mouse brains (Table 1). DHA and OA were detected only as free fatty acids, and no fatty acid was detected as fragment from LPC or PC.


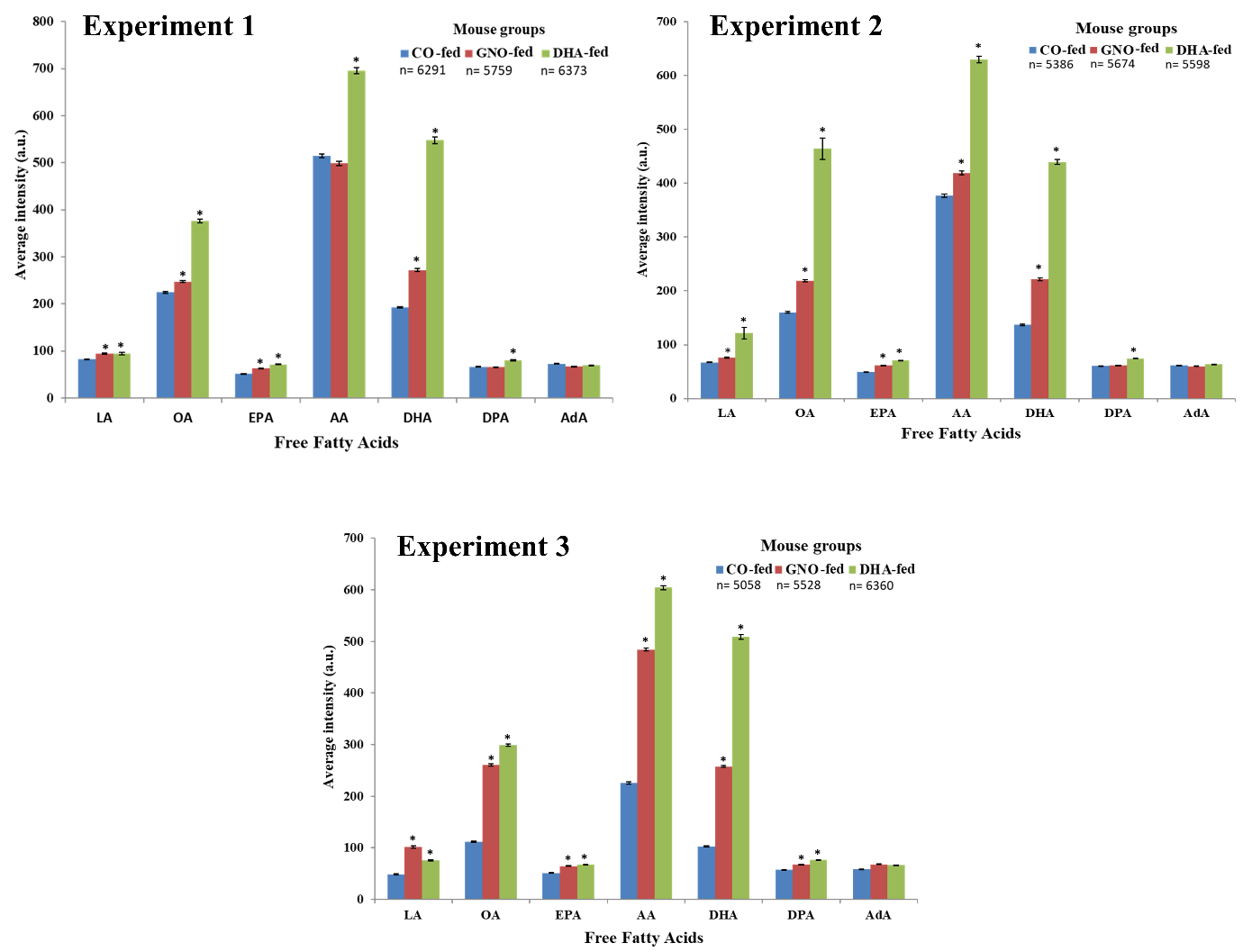


**Figure S4:** Average intensity (a.u.) of DHA and other free fatty acids in the sagittal slices of SAMP8 mouse brains from (data from 3 experiments). Here all values are expressed as mean ± SEM (**p* < 0.001 compared to CO), and “n” indicates number of pixels.


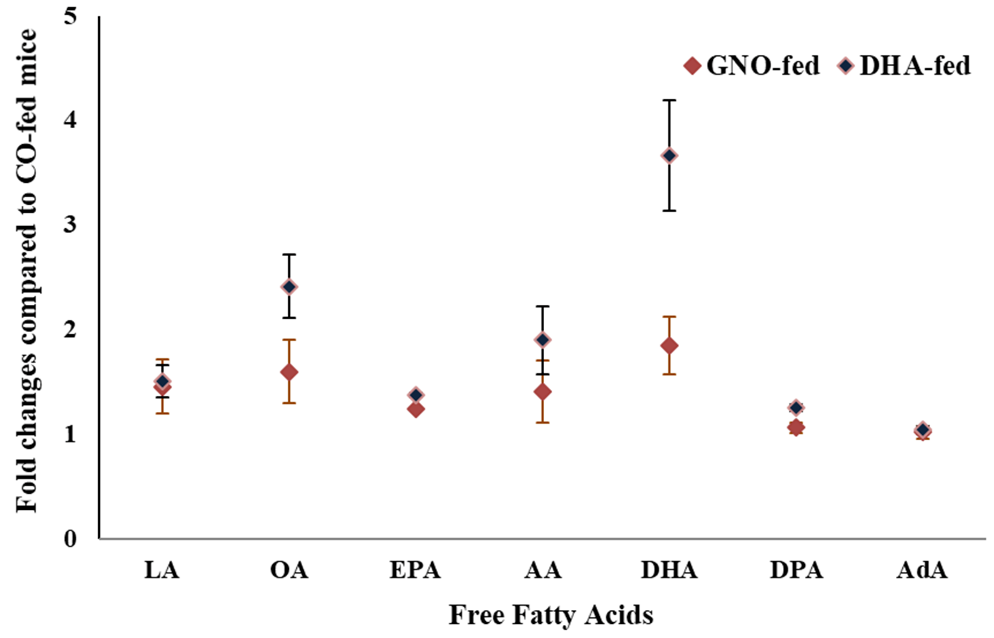


**Figure S5**: Fold changes in the distribution of free fatty acids in the brains of GNO-fed and DHA-fed SAMP8 mice compared to those of CO-fed SAMP8 mice. All data are presented as mean ± SEM (n=3; number of mice).

**Table S1**: Fold changes in the distribution of DHA in the different brain regions of DHA-fed and GNO-fed SAMP8 mice compared to CO-fed SAMP8 after the supplementation of CO, GNO and DHA.

| **Brain Regions** | **Mouse groups** | |
| --- | --- | --- |
|  | **GNO-fed** | **DHA-fed** |
| Cerebellum (Cb) | 1.86 ± 0.50 | 4.32 ± 1.46 |
| Cerebral Cortex (Cx) | 2.06 ± 0.86 | 3.55 ± 0.87 |
| Hippocampus (Hip) | 1.86 ± 0.52 | 3.39 ± 0.27 |
| Olfactory Bulb (OB) | 2.14 ± 0.24 | 4.19 ± 1.70 |
| Thalamus (Tha) | 2.00 ± 0.80 | 3.32 ± 0.51 |
| Hypothalamus (Hy) | 1.90 ± 0.30 | 2.33 ± 0.27 |
| Septum (ST) | 1.61 ± 0.78 | 2.78 ± 0.55 |

All values are expressed as Mean ± SEM (*n=3*; mice number).


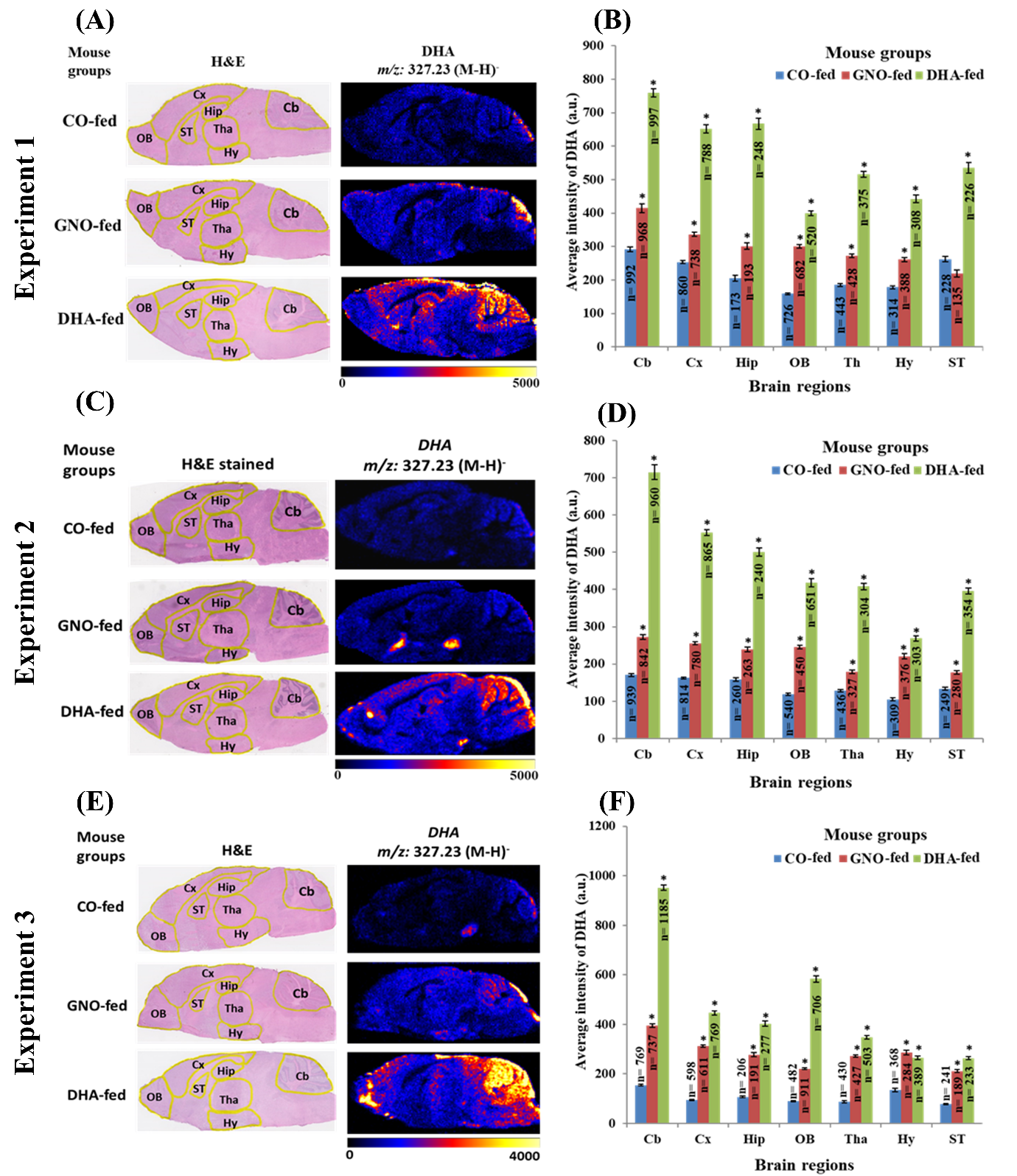


**Figure S6**: Distribution of DHA in different regions of SAMP8 mouse brains (data from three experiments). Figure (A), (C), and (E) represent spatial distributions of DHA in complete sagittal slices of SAMP8 mouse brains. Figure (B), (D), and (F) represent average intensity of DHA in different areas of SAMP8 mouse brains (**p* < 0.001 compared to CO). All values are expressed as mean ± SEM. Cb: cerebellum, Cx: cerebral cortex, Hip: hippocampus, OB: olfactory bulb, Tha: thalamus, Hy: hypothalamus, ST: septum, n: number of pixels.


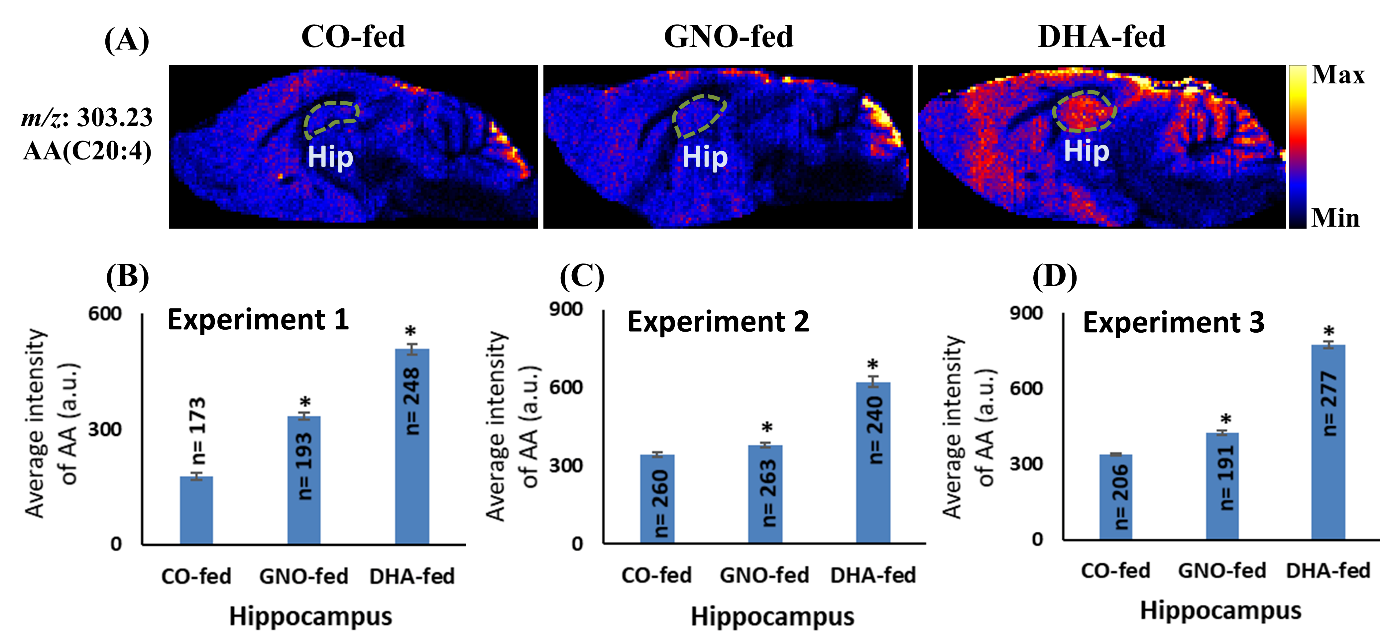


**Figure S7:** Distribution of arachidonic acid (AA) in the hippocampus of SAMP8 mice.

Figure (A) represents representative ion images of AA in the brain of SAMP8 mice treated with CO, GNO and DHA.

Figures (B-D) represent the average intensity of AA in the hippocampus of SAMP8 mice treated with CO, GNO and DHA from three experiments (**p* < 0.01 compared to CO). All values are expressed as mean ± SEM. Hip: hippocampus, n: number of pixels.
