# Supplementary material for: Dietary Intake of Green Nut Oil or DHA Ameliorates DHA Distribution in the Brain of a Mouse Model of Dementia Accompanied by Memory Recovery
Source: Nutrients. 2019 Oct 4;11(10):2371. doi: 10.3390/nu11102371 (PMC6835595; doi:10.3390/nu11102371)
Supplement: Supplementary file 1 [file nutrients-11-02371-s001.zip › Supplementary files/Table S1.docx]

**Table S1:** Fold changes in the distribution of DHA in the different brain regions of DHA-fed and GNO-fed SAMP8 mice compared to CO-fed SAMP8 after the supplementation of CO, GNO and DHA.

| **Brain Regions** | **Mouse groups** | |
| --- | --- | --- |
|  | **GNO-fed** | **DHA-fed** |
| Cerebellum (Cb) | 1.86 ± 0.50 | 4.32 ± 1.46 |
| Cerebral Cortex (Cx) | 2.06 ± 0.86 | 3.55 ± 0.87 |
| Hippocampus (Hip) | 1.86 ± 0.52 | 3.39 ± 0.27 |
| Olfactory Bulb (OB) | 2.14 ± 0.24 | 4.19 ± 1.70 |
| Thalamus (Tha) | 2.00 ± 0.80 | 3.32 ± 0.51 |
| Hypothalamus (Hy) | 1.90 ± 0.30 | 2.33 ± 0.27 |
| Septum (ST) | 1.61 ± 0.78 | 2.78 ± 0.55 |

All values are expressed as Mean ± SEM (*n=3;* mice number).
