# Supplementary figures and images for: Dietary Intake of Green Nut Oil or DHA Ameliorates DHA Distribution in the Brain of a Mouse Model of Dementia Accompanied by Memory Recovery
Source: Nutrients. 2019 Oct 4;11(10):2371. doi: 10.3390/nu11102371 (PMC6835595; doi:10.3390/nu11102371)

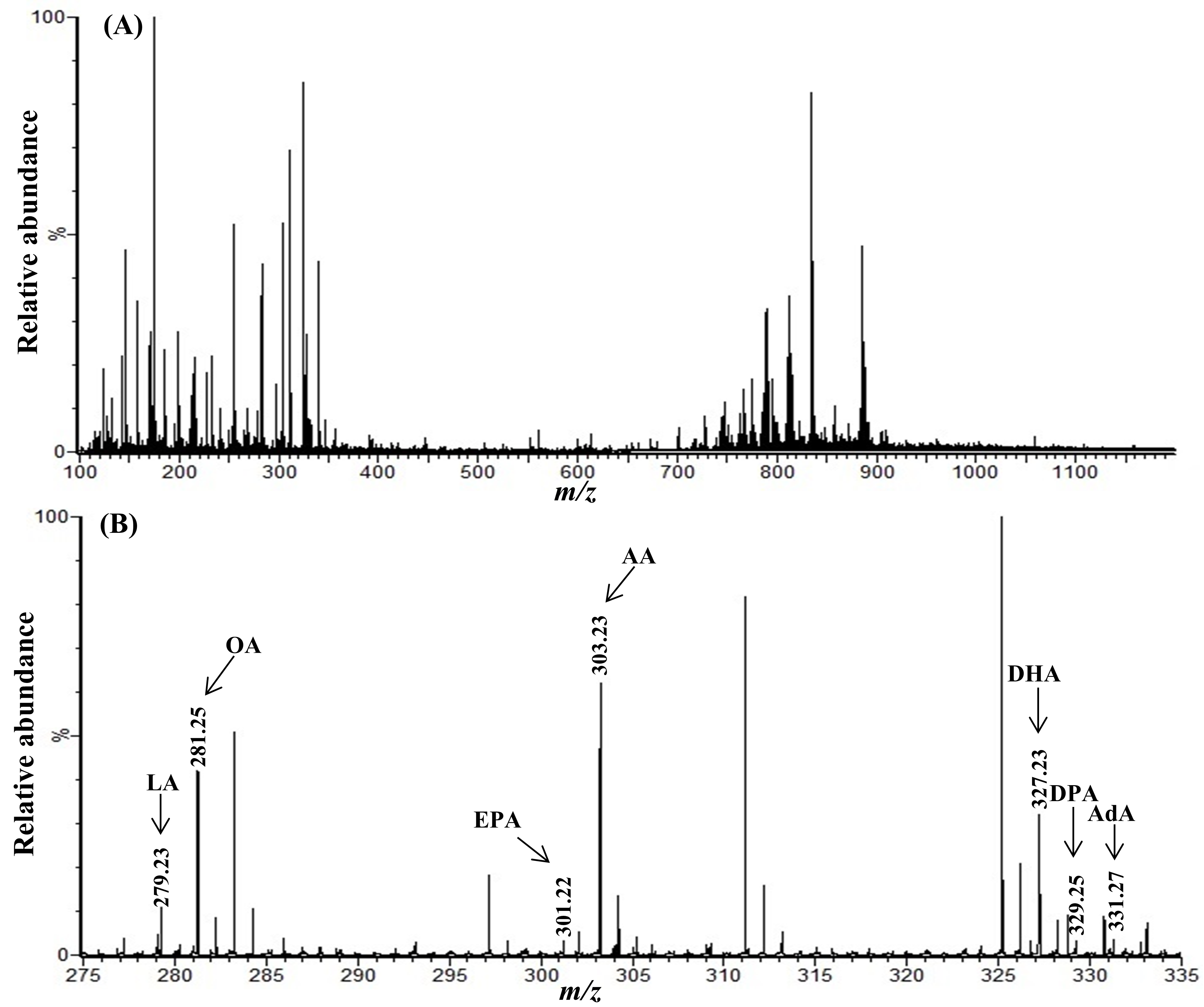

Supplement: Supplementary file 1 [file nutrients-11-02371-s001.zip › Supplementary files/Figure S1.tif]

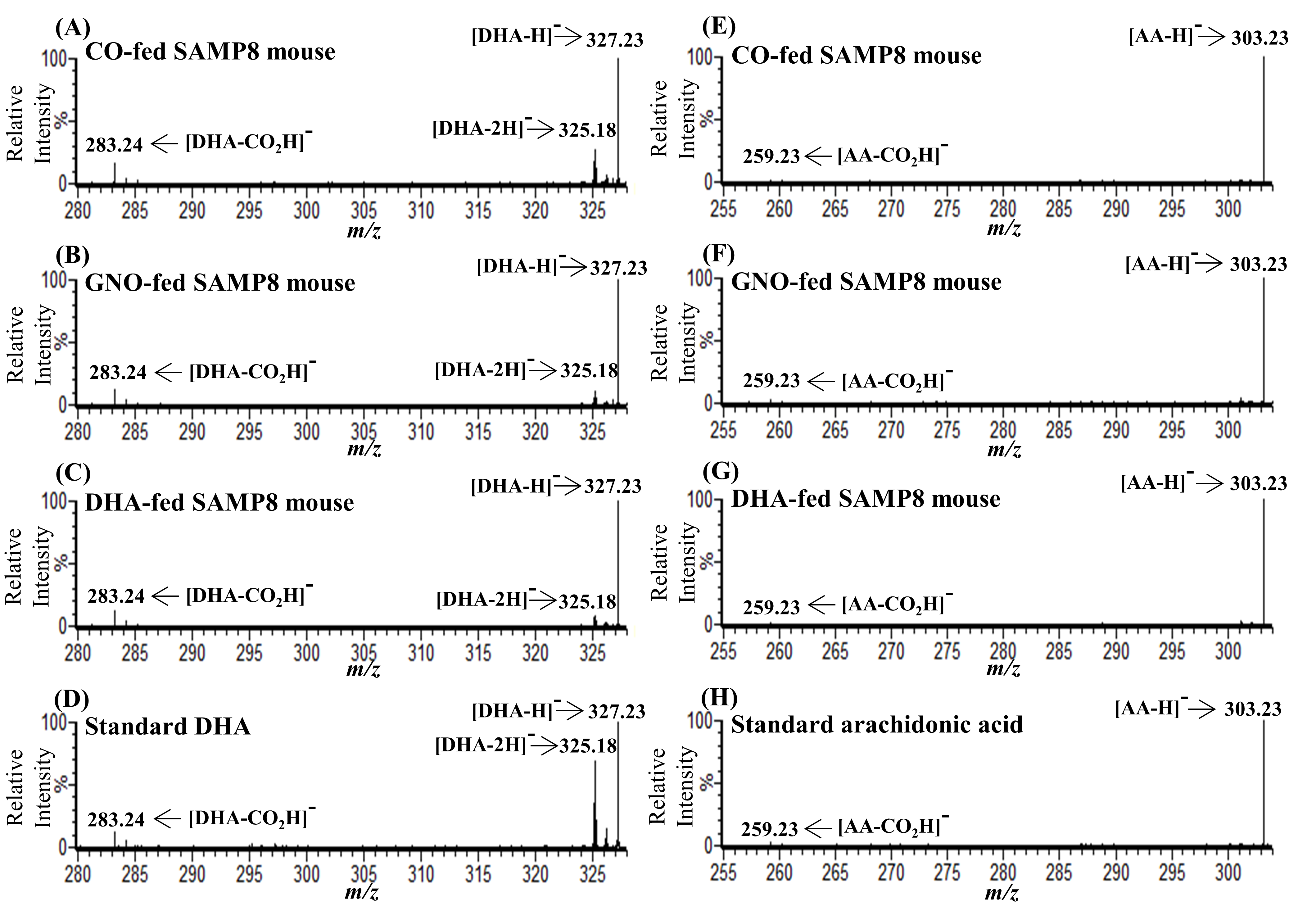

Supplement: Supplementary file 1 [file nutrients-11-02371-s001.zip › Supplementary files/Figure S2.tif]

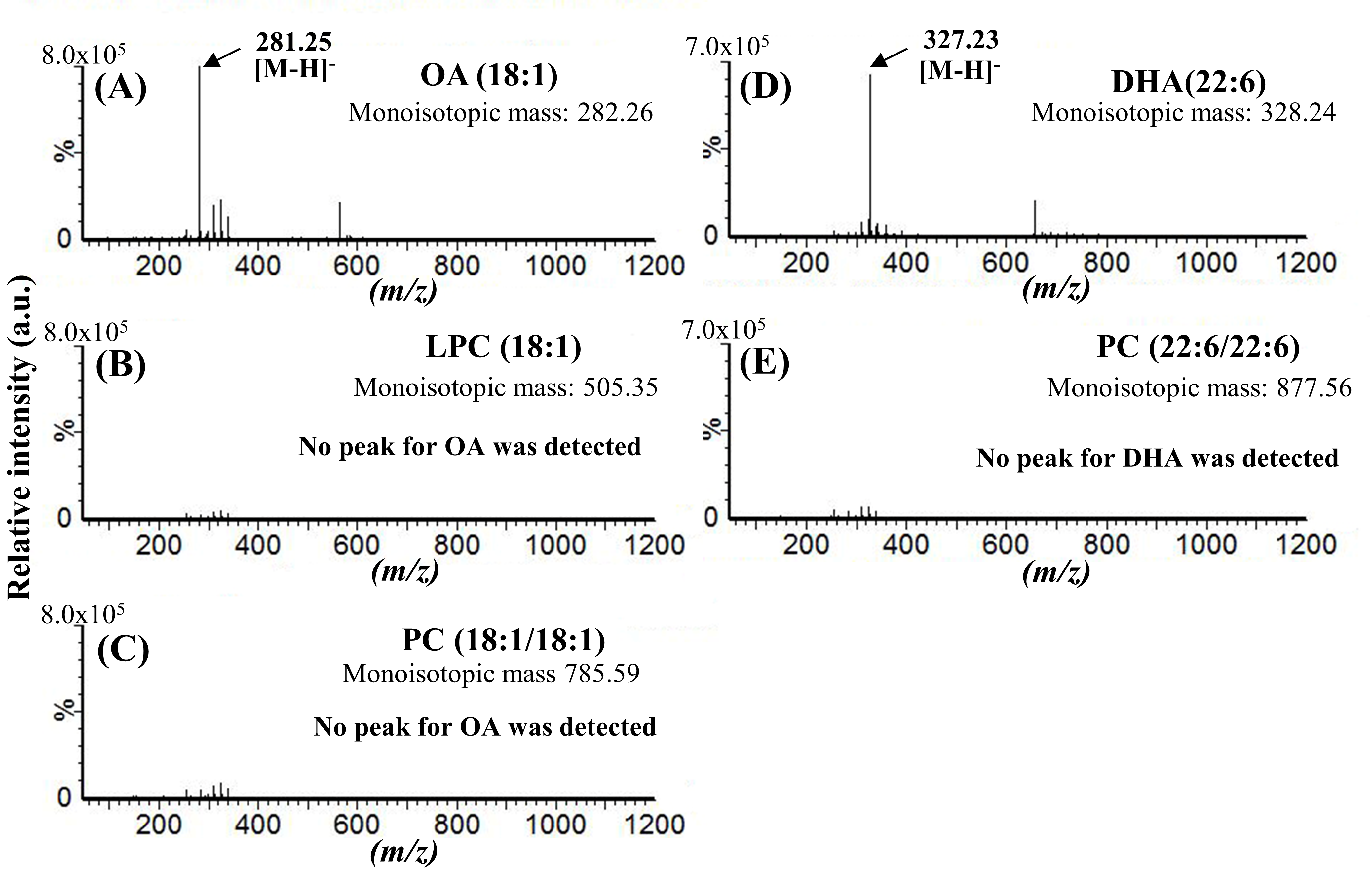

Supplement: Supplementary file 1 [file nutrients-11-02371-s001.zip › Supplementary files/Figure S3.tif]

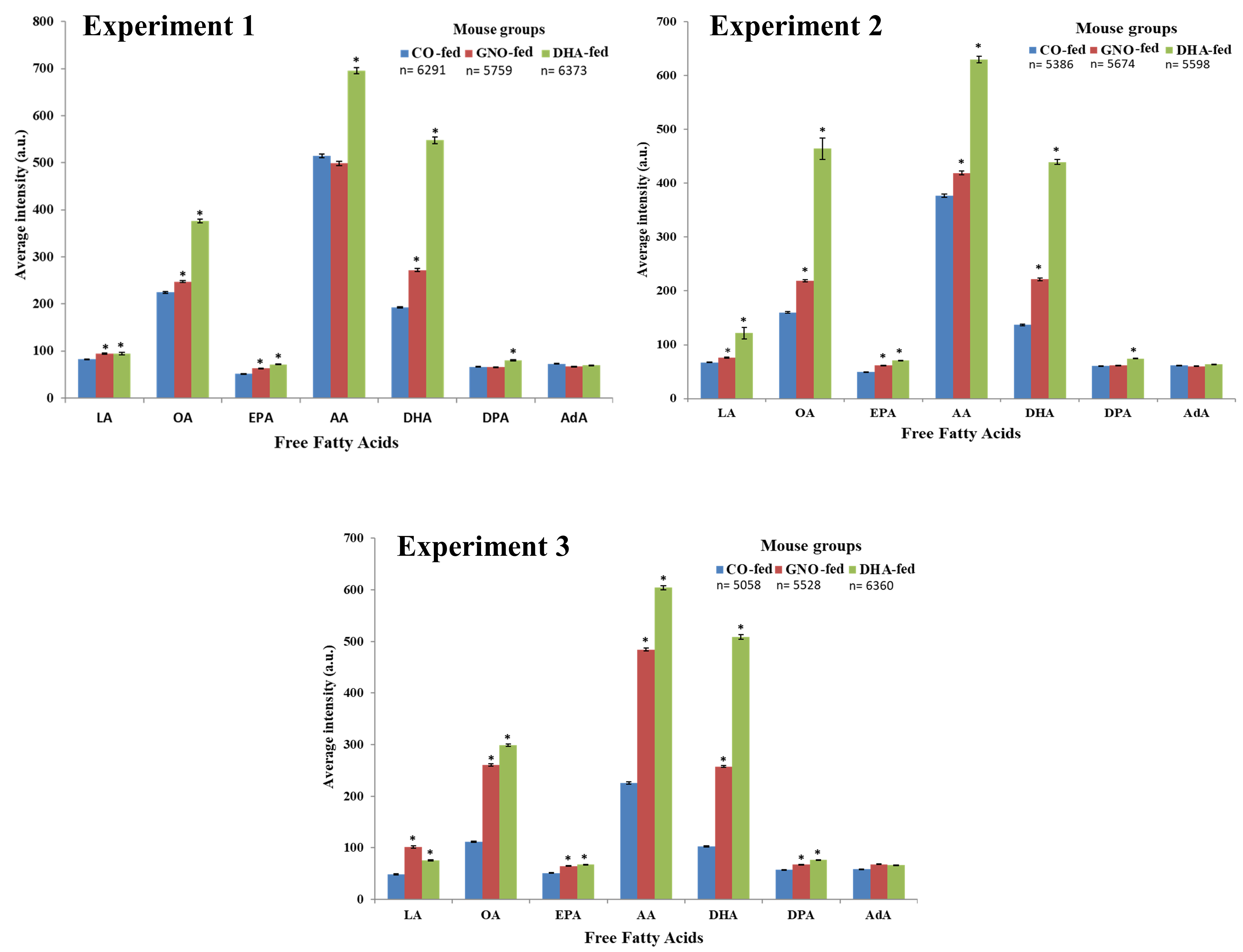

Supplement: Supplementary file 1 [file nutrients-11-02371-s001.zip › Supplementary files/Figure S4.tif]

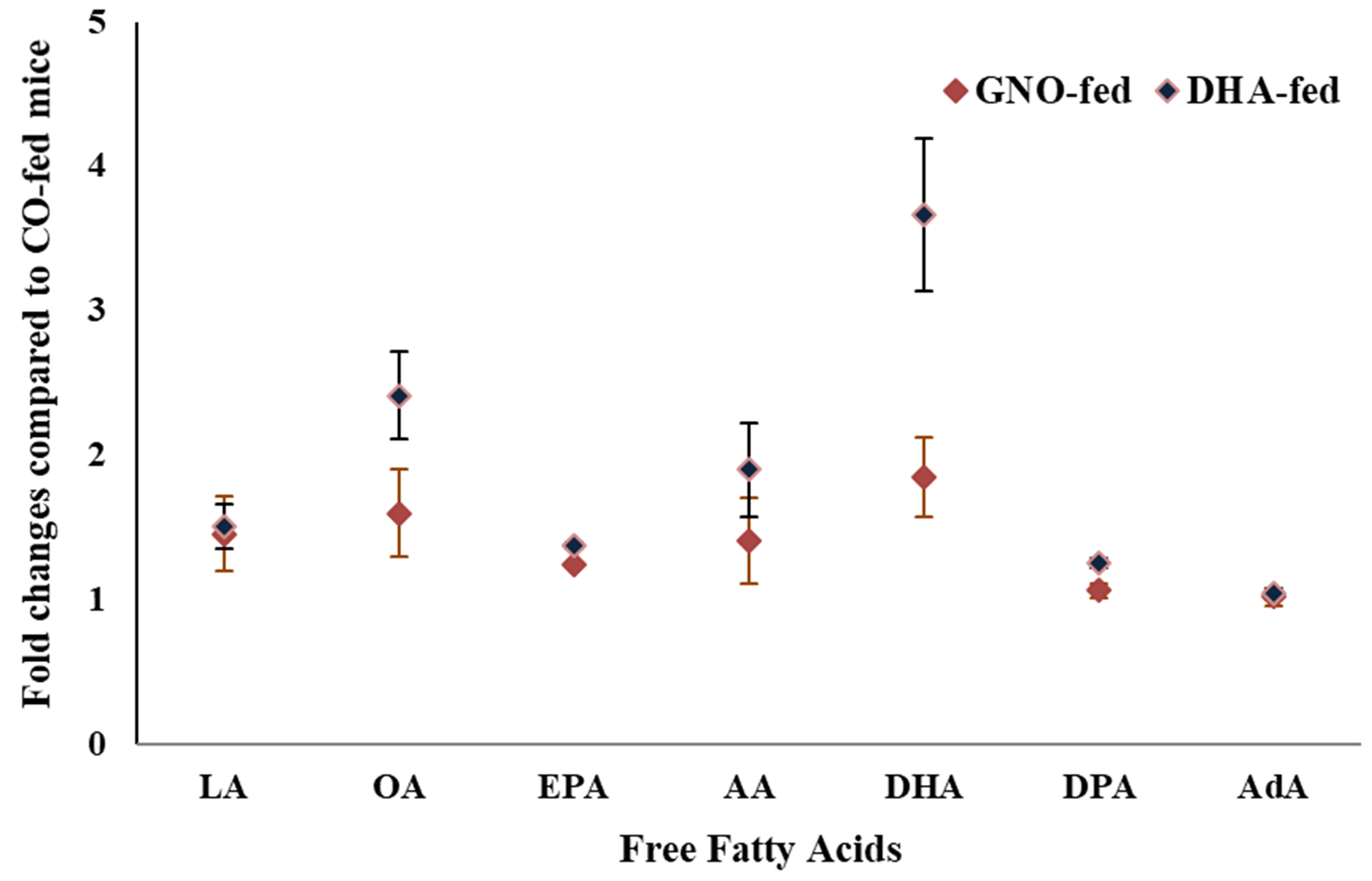

Supplement: Supplementary file 1 [file nutrients-11-02371-s001.zip › Supplementary files/Figure S5.tif]

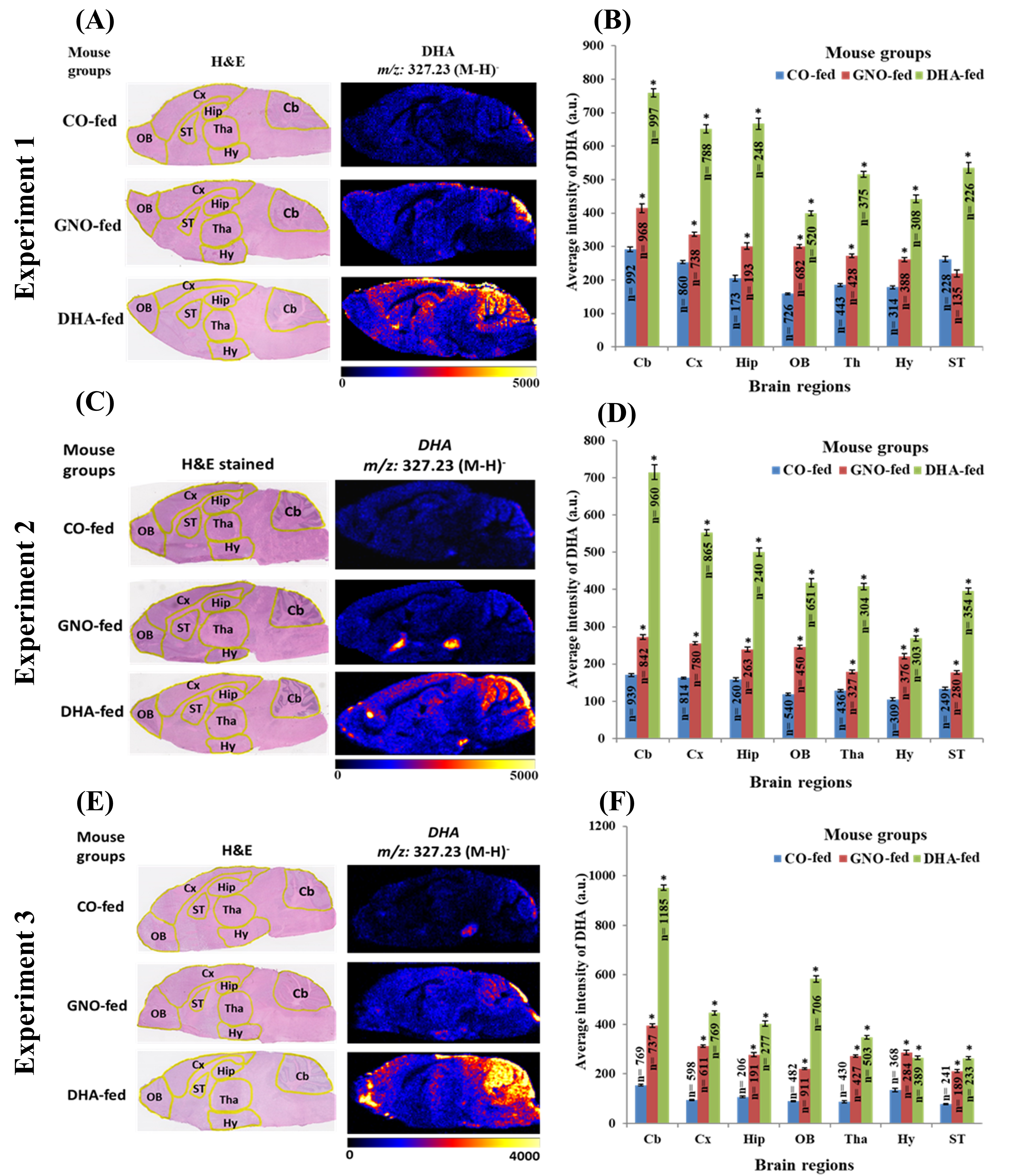

Supplement: Supplementary file 1 [file nutrients-11-02371-s001.zip › Supplementary files/Figure S6.tif]

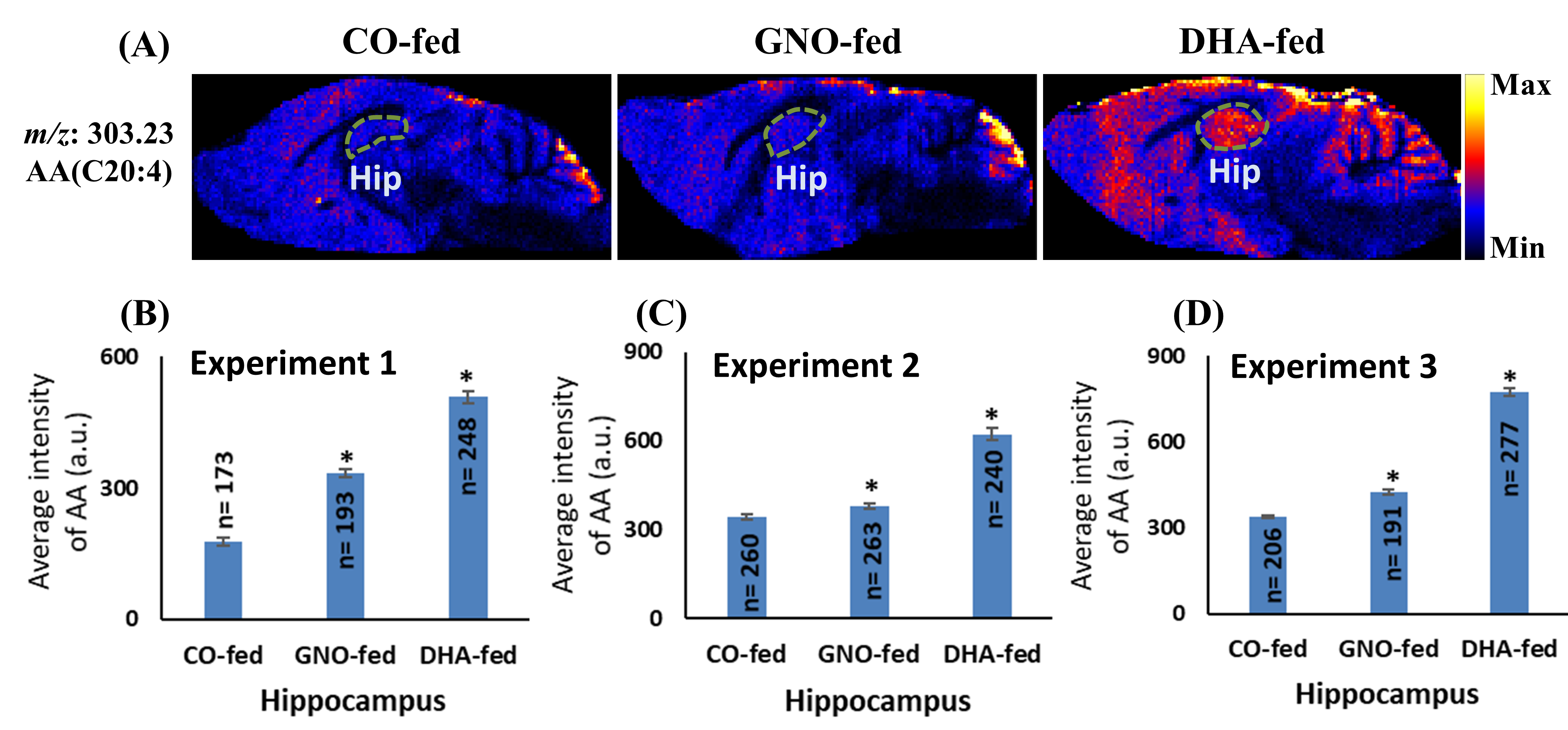

Supplement: Supplementary file 1 [file nutrients-11-02371-s001.zip › Supplementary files/Figure S7.tif]
